# Plasma/Serum Oxidant Parameters in Systemic Lupus Erythematosus Patients: A Systematic Review and Meta-Analysis

**DOI:** 10.1155/2024/9948612

**Published:** 2024-07-20

**Authors:** Napoleon Bellua Sam, Stephen Tabiri, Ebenezer Amofa

**Affiliations:** ^1^ Department of Medical Research and Innovation School of Medicine University for Development Studies, Tamale, Northern Region, Ghana; ^2^ Department of Surgery School of Medicine University for Development Studies, Tamale, Northern Region, Ghana; ^3^ Department of Physiology School of Medicine University for Development Studies, Tamale, Northern Region, Ghana

## Abstract

Most published results have revealed variations in the association of serum/plasma levels of malondialdehyde (MDA), apolipoprotein B (ApoB), and oxidized low-density lipoprotein (OxLDL) and systemic lupus erythematosus (SLE). This study was performed to establish MDA, ApoB, and OxLDL levels in systemic lupus erythematosus (SLE) patients. Electronic databases were searched for the included articles up to 27th February 2023. The meta-analysis included 48 articles with 2358 SLE patients and 2126 healthy controls considered for MDA, ApoB, and OxLDL levels. There were significantly higher MDA, ApoB, and OxLDL levels in SLE patients than those in the control groups. Subgroup analysis indicated that European/American SLE patients and patients of both ages <36 and ≥36 exhibited higher MDA, ApoB, and OxLDL levels. Arab and Asian SLE patients had higher ApoB and MDA/OxLDL levels. African SLE patients recorded higher OxLDL levels than the control groups. SLE patients with a body mass index (BMI) of ≥23 and a disease duration of <10 recorded significantly higher MDA, ApoB, and OxLDL levels. Patients with systemic lupus erythematosus disease activity index (SLEDAI) ≥8 of SLE had higher MDA and ApoB levels, whereas SLE patients with SLEDAI <8 showed significantly higher ApoB levels. Patients with BMI <23 of SLE had higher MDA and OxLDL levels. This study established significantly higher MDA, ApoB, and OxLDL levels in SLE patients, suggesting a possible role of MDA, ApoB, and OxLDL in the disease.

## 1. Introduction

Systemic lupus erythematosus (SLE) is a common complex autoimmune disease that is inflammatory, relapsing, and chronic. Although the exact cause of systemic lupus erythematosus (SLE) is still unknown, it is widely accepted that the autoimmune disease is marked by high autoantibody production [1], immune complexes, and compromised cellular and humoral immunity. These factors are most likely a result of a combination of genetic, environmental (virus, food, and UV light), and hormonal factors. In human and animal models, oxidative stress and the associated cellular damage have been implicated as key players in the etiology, development, progression, and maintenance of autoimmune disease [2–5]. Lipid peroxidation produces malondialdehyde (MDA), which can form intra- and intermolecular adducts with proteins by covalent bonding [6, 7].

MDA can react with nucleophilic amino acids such as cysteine and lysine, forming covalent MDA-protein adducts. MDA contains two aldehyde sets. Increased physiological disturbances, such as loss of structure and function, are brought on by oxidized proteins [8–10]. Oxidatively altered proteins cause further suffering in autoimmune disorders because they breach B-cell tolerance, making them possible targets for the immune system [11]. A prior study [12] had demonstrated that SLE patients experienced oxidative damage in addition to a markedly elevated conjugated diene and MDA production. Some studies [12, 13] also discovered that the antioxidant enzymes superoxide dismutase and catalase were the targets of increased levels of circulating autoantibodies in the plasma and serum of SLE patients.

A higher sensitivity of plasma/serum lipids and lipoproteins to oxidation may, at the very least, promote atherogenesis. SLE has been linked to early atherosclerosis [14]. The production of foam cells originating from macrophages may be intimately associated with the modification of low-density lipoprotein (LDL), a sort of modified LDL, that is triggered by scavenger receptors on macrophages rather than LDL receptors [15]. Thus, OxLDL has a major role in the development and course of atherosclerosis [16, 17]. Furthermore, OxLDL has been demonstrated to be a cardiovascular risk factor in people with SLE [18, 19].

Apolipoprotein B (ApoB) comes in two isoforms (ApoB48 and ApoB100) in human plasma and serum, and its levels are correlated with the risk of coronary heart disease. The primary physiological ligand for both the LDL receptor and the massive monomeric protein ApoB100, which has 4563 amino acids, is found in both. The liver produces ApoB100, which is necessary for the synthesis of extremely low-density lipoprotein. Unlike the other apolipoproteins, ApoB does not switch between lipoprotein particles; instead, it is present in LDL and intermediate density lipoprotein following the loss of ApoA, E, and C. Chylomicrons and their remnants include ApoB48. It is necessary for dietary lipids to be absorbed via the gut wall [20].

In the past years, various studies have shown plasma/serum MDA, ApoB, and OxLDL levels in SLE patients who also have established variations in plasma/serum MDA, ApoB, and OxLDL levels in SLE patients but with a smaller sample size [21–27]. However, the differences in studies go far beyond the explanations of standard units, ethnicity, and varied features of control groups. A systematic review and meta-analysis are conducted to find a distinct estimation of the relationship between serum or plasma MDA, ApoB, and OxLDL levels and SLE. This is performed in order to compare the plasma/serum oxidant status of SLE patients with the control groups and also to determine the likely role of MDA, ApoB, and OxLDL in the pathophysiology of SLE.

## 2. Materials and Methods

### 2.1. Systematic Search

Using PubMed, Embase, Web of Science, and ScienceDirect, a comprehensive search of published articles up to February 27, 2023, that documented the relationship between MDA, ApoB, and OxLDL levels and SLE was conducted. Malondialdehyde, MDA, Apolipoprotein, ApoB, Oxidized low-density lipoprotein, OxLDL, oxidative stress, systemic lupus erythematosus, and SLE were the search terms utilized. In order to locate other relevant papers that were not electronically collected by the aforementioned databases, the references of reviews and retrieved articles were also searched. E-mail requests for missing data were made to the respective authors.

### 2.2. Criteria for Inclusion

When there was uncertainty or disagreement between the two researchers regarding the eligibility of a particular article for inclusion, a third researcher was asked to mediate. Two researchers from the research team evaluated all retrieved articles independently to determine whether each article met the requirements for inclusion. The only human subjects, plasma/serum (MDA, ApoB, and OxLDL) levels, original data, no language or racial/ethnicity restrictions, and case-control study, cross-sectional study, and cohort study were the inclusion criteria for this meta-analysis. To ensure inclusion, articles containing values in the range, median, standard error, interquartile range, and mean were transformed to mean and standard deviation [28]. When the same data were provided in many articles, the most recent, thorough data were chosen.

### 2.3. Exclusion of Articles

We excluded articles with insufficient data, reviews, and case reports in the meta-analysis. All original articles on animals as well as duplicate articles were excluded in the analysis. The lack of estimates for the variables taken into account for the subgroup analysis was not the basis for the exclusion criteria. For each individual publication, a record of the reasons as to why it was excluded from the study was established.

### 2.4. Extraction of Data

From the included articles, we extracted variables such as first author, year, country, number, age (mean ± SD), gender, disease period, body mass index (BMI), systemic lupus erythematosus disease activity index (SLEDAI), MDA assay, ApoB assay, OxLDL assay, ethnicity, study type, and MDA levels (mean ± SD), ApoB levels (mean ± SD) and OxLDL levels (mean ± SD). Only data extraction from original publications was performed by two different researchers. Also, from the included articles, the following covariate variables: country, publication year, sample size, study type, ethnicity, measurement type, and NOS, were extracted based on literature to conduct the meta-regression. The confounding factors were selected based on an extensive review of the literature and were chosen according to the common factors identified across various studies. The Preferred Reporting Items for Systematic Reviews and Meta-Analysis (PRISMA) standards [29] were followed in this analysis, and the Newcastle–Ottawa Quality Assessment Scale (NOS) [30] was used to evaluate the methodological quality of the included papers. A technique quality score of ≥5 to 9 denoted superior quality for the considered article.

### 2.5. Statistical Analysis

Since we let the genuine effects to vary between studies, the random effect model was used for the analysis. To obtain the estimated values in mean and standard deviation, the conversion was performed for all data presented as either median and range or median and interquartile range (IQR) [28]. Prior to analysis, articles that provided levels of ApoB, OxLDL, and plasma/serum MDA in units other than µg/ml were converted. For every investigation, the standard mean difference (SMD) and its 95% confidence interval (CI) were used to calculate the associations between plasma/serum MDA, ApoB, and OxLDL levels and SLE. The pooled SMD and 95% CIs were tested for significance using the *Z*-test; a value of *P* < 0.05 was considered statistically significant.

Stratified analysis was performed to successfully perform subgroup analysis. Using Cochrane's *Q* statistics (*Q*-test), heterogeneity among the included articles was assessed; *P* ≤ 0.01 was deemed statistically significant [31, 32]. In addition, the *I*^2^ statistics (*I*^2^=(*Q* − df)/*Q* × 100%) were used to evaluate heterogeneity. According to reference [33], heterogeneity was indicated by an *I*^2^ value of less than 25%, moderate heterogeneity by *I*^2^ = 25 to 50%, significant heterogeneity by *I*^2^ > 50 to 75%, and extreme heterogeneity by *I*^2^ > 75%. We performed subgroup analysis to identify the sources of heterogeneity.

In addition, to identify the studies that overcontributed to the observed heterogeneity, sensitivity analysis was performed. When heterogeneity was observed, the source of the heterogeneity was found using meta-regression analysis. With Egger's linear regression and Begg's rank correlation tests, publication bias was evaluated [34]. When publication bias was likely, the trim and fill analysis was applied. When *P* < 0.05, the publication bias was deemed statistically significant. Each and every analysis was conducted using STATA version 12.

## 3. Results

### 3.1. Literature Search

**Figure 1:**
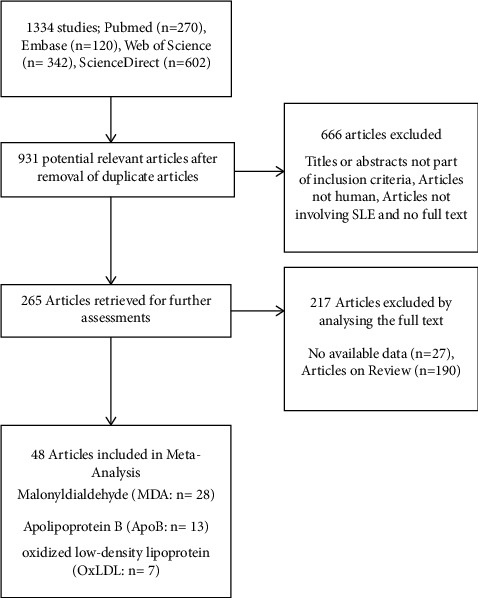
Flowchart of the exclusion process of articles and the reasons.

A total of 1334 articles from PubMed, Embase, Web of Science, and numerous ScienceDirect were obtained, with retrievals of 270, 120, 342, and 602 in that order. Following a screening process, 403 duplicate articles and 666 papers lacking an abstract or related title (not pertaining to people, full text, or SLE) were eliminated from the analysis. After more thorough evaluations of the 265 remaining publications, 217 of them (of which 27 contained no data at all and 190 were merely reviewed papers) were eliminated, leaving 48 articles for this meta-analysis [12, 21–27, 35–64]. Among the 48 included articles on SLE, 28, 13, and 7 were from MDA [12, 26, 27, 35–54], ApoB [21, 22, 55–62], and OxLDL [23–25, 55, 63–65], respectively (Figure 1).

### 3.2. Study Characteristics

This meta-analysis included forty-eight articles with 2358 patients of SLE and 2126 health controls assessed for MDA (SLE: 1041 and control: 1041), ApoB (SLE: 853 and control: 834), and OxLDL (SLE: 464 and control: 834) levels. One article out of every sixteen was from a cross-sectional study, while nearly 45 percent (43.8%) of the included papers used cohort studies as their study type. In precisely half of the included papers, case-control studies were used. The European/American ethnicity was categorized by Hungarian, North American, South American, German, Caucasian, Sweden, and mixed ethnic groups. Colorimetric assay, thiobarbituric acid reactive species, microplate reader, fluorometric assay, spectrophotometric method of lipid peroxidase LPO-586, high-performance liquid chromatography, chemiluminescent assay, immunoturbidimetric assays, nephelometric method, and radial immunodiffusion were the measurement methods/types employed for plasma/serum MDA, ApoB, and OxLDL levels. The assessment of the quality of the methodology was satisfactory for all the included articles with ratings between five and nine.  Table 1 lists the specific characteristics of the articles that are included.

### 3.3. Meta-Analysis Results

#### 3.3.1. Heterogeneity Results

Heterogeneity was significant throughout the studies (all *P* < 0.001, Table 2).

#### 3.3.2. Plasma/Serum MDA, ApoB, and OxLDL Levels

**Figure 2:**
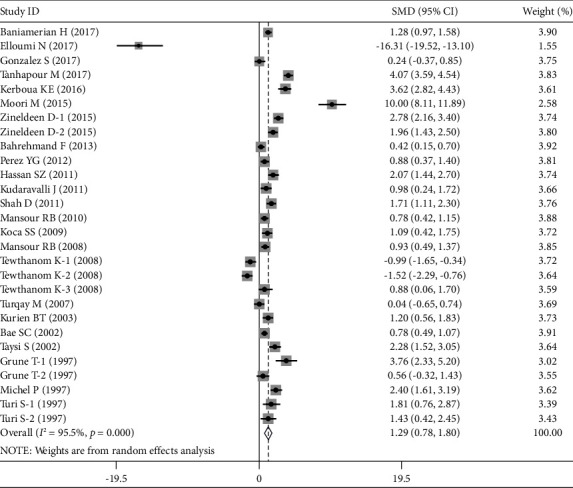
Forest plots of the pooled odd ratio and 95% confidence intervals of included articles and relationship for MDA levels and SLE.

**Figure 3:**
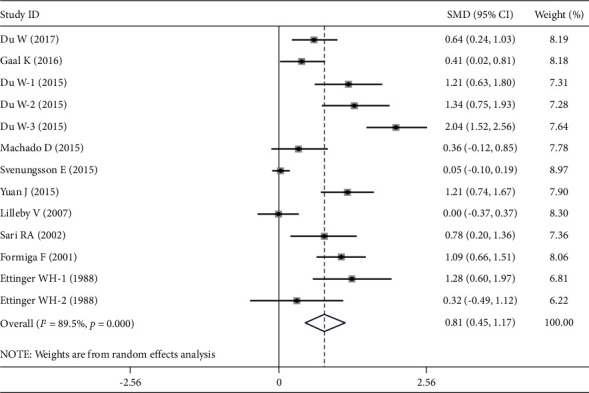
Forest plots of the pooled odd ratio and 95% confidence intervals of included articles and relationship for ApoB levels and SLE.

**Figure 4:**
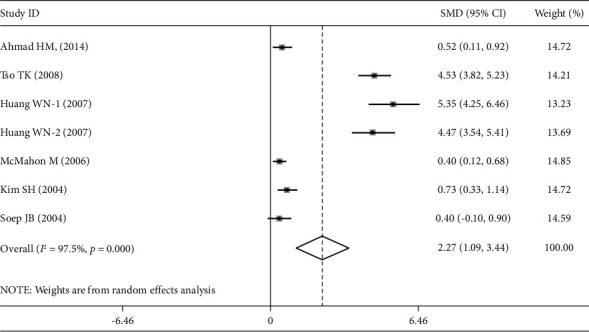
Forest plots of the pooled odd ratio and 95% confidence intervals of included articles and relationship for OxLDL levels and SLE.

Compared to control groups, plasma/serum MDA levels were considerably higher in SLE patients (SMD = 1.292 ng/ml; 95% CI = 0.780–1.805; *Z* = 4.94; *P* < 0.001). In addition, the plasma/serum ApoB levels of SLE patients were significantly higher than those of the control groups (SMD = 0.808 ng/ml; 95% CI = 0.451–1.165; *Z* = 4.44; *P* < 0.001). Once more, compared to control groups, SLE patients' plasma/serum OxLDL levels were significantly higher (SMD = 2.266 ng/ml; 95% CI = 1.088–3.444; *Z* = 3.77; *P* < 0.001). Figures 2, 3, and 4 show the pooled results for the levels of MDA, ApoB, and OxLDL, respectively. These results suggest that MDA may have a role in the oxidative transformation of LDL particles, which results in the production of OxLDL. Patients with SLE may have elevated levels of OxLDL, which can worsen inflammation and have a role in the development of atherosclerosis and other cardiovascular problems. Since ApoB is an LDL component, it affects how OxLDL is transported and metabolized in SLE.

### 3.4. Subgroup Analysis

The variables that were taken into account for the subgroup analysis were measurement type, age, BMI, SLEDAI, disease duration, and ethnicity. The results are shown in Table 3. It was established that European/American SLE patients as well as patients of both ages <36 and ≥36 exhibited higher MDA, ApoB, and OxLDL levels when compared with the control groups. It was shown that Arab and Asian SLE patients had higher ApoB and MDA/OxLDL levels as compared with the controls. Also, it was evident that African SLE patients recorded higher OxLDL levels than the control groups. SLE patients with a body mass index (BMI) of ≥23 as well as a disease duration of <10 recorded significantly higher MDA, ApoB, and OxLDL levels compared to the control groups. It was further revealed that patients with SLEDAI ≥8 of SLE had higher MDA and ApoB levels compared to the controls and SLE patients with SLEDAI score of <8 showed significantly higher ApoB levels. Patients with a BMI of <23 of SLE had higher MDA and OxLDL levels and also with disease duration ≥10 (SMD = 2.458 ng/ml; 95% CI = 1.011–3.905; *Z* = 3.33; *P* ≤ 0.01) of SLE had significantly higher MDA levels.

### 3.5. Publication Bias and Sensitivity Analysis

Egger's test revealed a substantial publishing bias in all the included individual publications for SLE and ApoB (*P* < 0.05; *t* = 3.77; Table 2) and OxLDL (*P* < 0.05; *t* = 4.41; Table 2), while Begg's test revealed no significant publication bias throughout the included articles (*P* > 0.05; Table 2).

**Figure 5:**
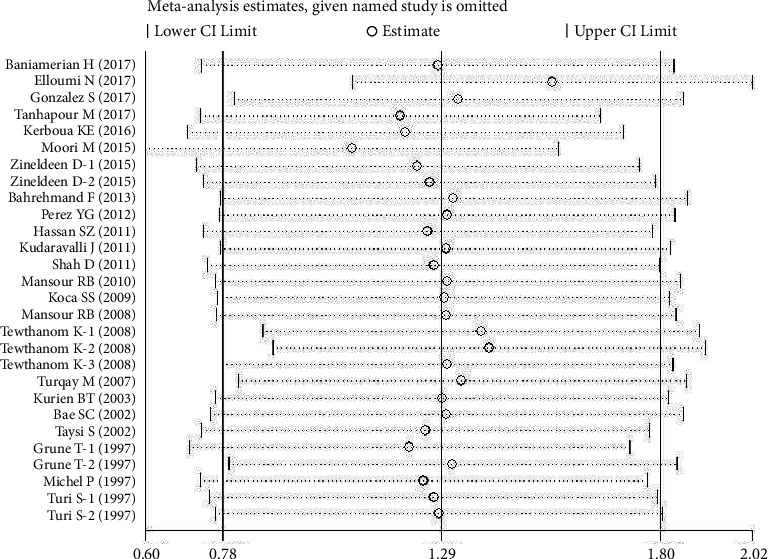
Sensitivity analysis of the included articles for MDA levels. The two vertical axes, vertical middle axis, hollow circles, and two ends of the dotted lines, respectively, represent, overall odd ratio, pooled odd ratios, and 95% confidence interval.

**Figure 6:**
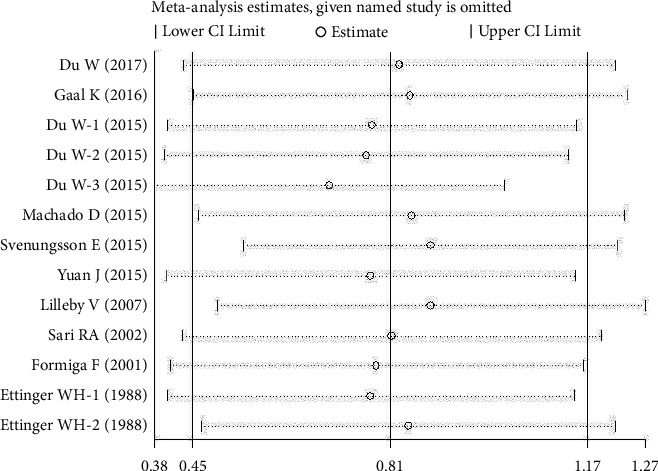
Sensitivity analysis of the included articles for ApoB levels. The two vertical axes, vertical middle axis, hollow circles, and two ends of the dotted lines, respectively, represent, overall odd ratio, pooled odd ratios, and 95% confidence interval.

**Figure 7:**
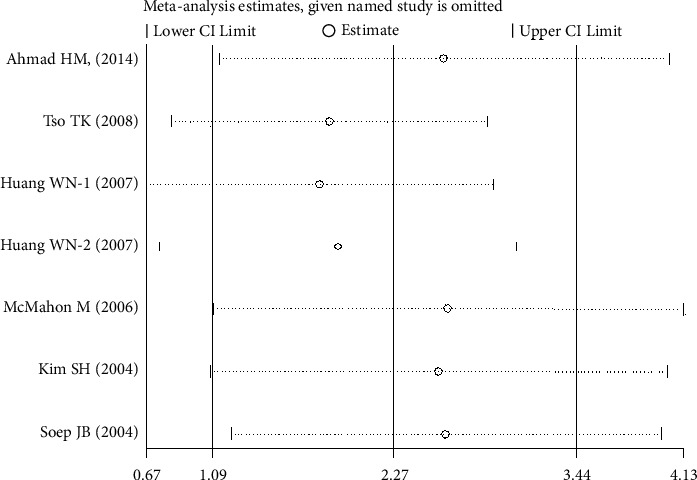
Sensitivity analysis of the included articles for OxLDL levels. The two vertical axes, vertical middle axis, hollow circles, and two ends of the dotted lines, respectively, represent, overall odd ratio, pooled odd ratios, and 95% confidence interval.

However, there was no significant publication bias shown by Egger's test in all the included articles for SLE and MDA (*P* > 0.05; *t* = 0.65; Table 2). Furthermore, trim and fill analysis was carried out for plasma/serum ApoB levels with *P* < 0.001, keeping the number of trials (13) the same. This demonstrated our consistent findings. Again, trim and fill analysis was performed for plasma/serum OxLDL levels and the results showed trimming with the number of studies changed from seven to eight. Moreover, *P*=0.002 shows that our results were steady. Sensitivity analysis did not significantly alter the pooled results, suggesting the results to be stable (Figures 5, 6, and 7).

### 3.6. Meta-Regression Analysis

A meta-regression analysis with the inclusion of covariate variables (country, publication year, sample size, study type, ethnicity, measurement type, and NOS) was carried out to find additional potential drivers of heterogeneity. According to the results in Table 4, all of the covariates had no effect on the overall effect size or on the correlation between plasma/serum MDA, ApoB, and OxLDL levels and SLE (all *P* > 0.05).

## 4. Discussion

This study largely evaluated a lot of literature on the association between plasma/serum MDA, ApoB, and OxLDL and SLE, in which a total of 48 articles were included. There was enough evidence to establish that significantly higher plasma/serum MDA, ApoB, and OxLDL levels were observed in SLE patients compared to control groups even though heterogeneity existed within the included studies. We performed subgroup analysis and meta-regression to find the sources of heterogeneity. Numerous clinical characteristics, including lupus nephritis and tissue damage in SLE, have been linked to elevated MDA levels [54, 66]. In a similar vein, Koca SS et al. discovered that SLE patients had higher MDA levels than control groups. On the other hand, Tewthanom et al. reported that there was no statistically significant difference between the MDA levels of patients with SLE and the control groups [27]. The results of the Frosegard group's research have demonstrated that elevated levels of autoantibodies and OxLDL are risk factors for cardiovascular disease in SLE patients [67]. Likewise, Kim SH et al. revealed a significantly higher relationship between OxLDL levels in SLE [24]. However, our meta-analysis result of OxLDL levels does not support the findings of Soep et al., who reported no statistical significance between SLE patients and the control group [25]. A study conducted by Du et al. is in support with our finding that sought to indicate elevated ApoB levels in SLE patients as compared to control groups [21]. The ApoB levels of SLE patients and control groups did not differ significantly, according to Yuan et al. [22]. These differences can be related to the number of studies, the inclusion criteria, the measurement techniques, and, in the main, the sample size used in the original publications. Oxidative stress parameters play a crucial role in forecasting the consequences of oxidation and in providing the foundation for developing an appropriate response that can prevent or mitigate harm. Numerous studies examining MDA, ApoB, and OxLDL levels in SLE have been conducted since the disease was shown to be multigenic, with a range of results. Our research revealed a relationship with susceptibility to SLE, which was highly beneficial in elucidating the pathophysiology of SLE. MDA, ApoB, and OxLDL may represent a new SLE disease marker.

Disease activity in SLE patients is correlated with elevated plasma/serum MDA levels [12, 47]. This confirms our findings that plasma/serum MDA levels were greater in patients with SLEDAI ≥8 of SLE. According to our research, there is a substantial correlation between SLE patients' SLEDAI scores and their plasma/serum ApoB levels. In addition, a subgroup study showed a significant correlation between age and the plasma/serum levels of MDA, ApoB, and OxLDL. Age and plasma/serum levels (MDA, ApoB, and OxLDL) do not appear to be significantly correlated in the majority of earlier research [21, 22, 25, 37, 38, 44, 50, 55, 58, 63]. Consistent with our results, Mcmahon et al. found a strong correlation between age and plasma/serum OxLDL [65], which may indicate that hormonal factors have a role in the pathophysiology of SLE patients, the majority of whom are female during the reproductive time [68]. The different measuring methods and small sample sizes used in each study may be the cause of the discrepancies in the results. Our study's findings also showed that, as compared to healthy controls, ethnicity was substantially correlated with the plasma/serum levels (MDA, ApoB, and OxLDL) in SLE patients. Nevertheless, Soep et al. found no evidence of a connection between OxLDL levels and SLE [25]. The study's variability could be ascribed to specimen source, genetic variables, and ethnic differences. Since the majority of individual articles were written by people of Asian, European, and American ethnic backgrounds, more research from diverse geographic locations and ethnic groups or nations examining the plasma/serum levels of MDA, ApoB, and OxLDL in SLE patients compared with health controls may provide insight on the understanding of the pathophysiology of SLE. Our research revealed a strong correlation between the BMI of SLE patients and their plasma/serum levels (MDA, ApoB, and OxLDL), which contradicts the findings of [25, 37, 44, 50, 55, 58, 62–64]. The primary causes of the discrepancy may be attributed to the quantity of research and the precise data extraction methods used in our investigation. This research also showed that, for comparison groups, individuals with SLE had significantly greater levels of plasma/serum MDA, ApoB, and OxLDL than patients in the control group. MDA levels and the length of the sickness were found to be significantly correlated by Zineldeen et al. [40]. However, Tewthanom et al. [27] and Hassan et al. [44] provided evidence to the contrary.

This study is known as the first meta-analysis to show that plasma/serum MDA, ApoB, and OxLDL levels were examined in SLE patients relative to healthy controls. In addition, the included papers were not limited based on language, and the results were extracted accurately and validly. In comparison to the individual publications, our study's statistical power and analytical resolution were enhanced by the utilization of a sizable sample size. In addition, sensitivity analysis, trim and fill, and meta-regression were used to find confounding variables, heterogeneity, and publication bias. Nonetheless, certain shortcomings were noted. Certain materials, such as academic dissertations and conference abstracts, were left out and could have skewed the actual results. Once more, just a small number of components' causes of heterogeneity were examined via subgroup analysis and meta-regression. The large degree of variability in this study may be explained by a few different factors, including drug use and other clinical characteristics.

## 5. Conclusion

Our research revealed that the levels of plasma/serum MDA, ApoB, and OxLDL were considerably higher in SLE patients than in the control group. This suggests that MDA, ApoB, and OxLDL are important factors to consider when analyzing oxidative stress and play a crucial role in the pathophysiology of SLE. Future research should examine bigger sample sizes while accounting for the environmental determinants of MDA, ApoB, and OxLDL levels in individuals with SLE.

## Figures and Tables

**Table 1:** Characteristics of individual studies included in the meta-analysis: (a) MDA levels and SLE. (b) ApoB levels and SLE. (c) OxLDL levels and SLE.

(a)																	
First author, year	Country	SLE					Control			Assay method, sample	Ethnicity	Study type	MDA level (SLE)		MDA level (control)		NOS
		*n*	Age (mean ± sd, years)	SLEDAI	BMI	Duration (mean ± sd, years)	*n*	Age (mean ± sd, years)	BMI				Mean	SD	Mean	SD	

Baniamerian 2017 [35]	Iran	100	37.1 ± 11.5	NA	NA	NA	98	35.6 ± 16.3	NA	HPLC	Arab	CC	21.6	11.6	10.7	3.2	7
Elloumi 2017 [36]	Tunisia	30	NA	≥6	NA	NA	23	30.6 ± 7.7	NA	TBARS	African	CC	1.73	0.06	3.12	0.11	7
Gonzalez 2017 [37]	Spain	21	48.14 ± 11.53	≤8	25.23 ± 4.87	9.81 ± 7.08	21	46.24 ± 9.45	25.72 ± 4.67	SMPLO	Caucasian	CC	2.87	0.42	2.77	0.41	8
Tanhapour 2017 [38]	Iran	107	35.6 ± 16.3	NA	NA	NA	101	37.1 ± 11.5	NA	HPLC	Arab	CC	1.84	0.26	1.04	0.09	7
Kerboua 2016 [39]	Algeria	16	27.86 ± 6.26	10 ± 2.3	NA	69.65 ± 54.65	60	27.88 ± 8.28	NA	TBARS	African	CC	613.00	56.21	460.00	37.85	7
Zineldeen 2016 [40]	Egypt	40	46.2 ± 5.28	12.9 ± 2.3	25.87 ± 1.8	10.5 ± 1.36	40	45.15 ± 5.35	24.9 ± 2.2	ELISA	African	CS	7.37	2.92	1.58	0.40	7
Zineldeen 2016 [40]	Egypt	40	44.7 ± 5.8	12.5 ± 4.8	24.89 ± 2.1	5.025 ± 1.61	40	45.15 ± 5.35	24.9 ± 2.2	ELISA	African	CS	6.08	3.22	1.58	0.40	7
Moori 2015 [41]	Iran	30	29.3 ± 5.28	NA	NA	NA	30	28.83 ± 6.47	NA	TBARS	Arab	CC	4.90	0.30	1.90	0.30	7
Bahrehmand 2013 [42]	Iran	109	35.6 ± 16.3	21 ± 12.2	NA	NA	103	37.1 ± 11.5	NA	HPLC	Arab	CC	18.40	12.70	14.10	6.40	8
Pérez 2012 [43]	Brazil	36	28.2 ± 13	10.3 ± 6.6	NA	5.9 ± 3.5	28	27.9 ± 9.9	NA	HPLC	SA	CC	3.90	2.60	1.60	2.60	7
Hassan 2011 [44]	Egypt	30	32.47 ± 10.89	12.8 ± 6.89	26.5 ± 5.82	6.58 ± 6.33	30	32.3 ± 6.73	27.38 ± 4.01	TBARS	African	CC	2.78	0.78	1.57	0.27	9
Kudaravalli 2011 [45]	India	32	29 ± 8	5 ± 2	NA	29 ± 10	10	24 ± 6	NA	NA	Asian	CC	11.15	3.08	8.25	2.50	5
Shah 2011 [46]	India	30	26.5 ± 7.48	35.56 ± 16.31	NA	5.1 ± 2.20	30	26.73 ± 5.37	NA	TBARS	Asian	CC	1.25	0.44	0.62	0.28	7
Mansour 2010 [47]	Tunisia	65	NA	NA	NA	NA	60	NA	NA	TBARS	African	CC	102.49	56.04	58.73	56.05	6
Koca 2009 [26]	Turkey	30	37.25 ± 11.03	12.5 ± 7.35	22.6 ± 3.14	5.03 ± 3.41	15	36.5 ± 10.35	23.98 ± 2.85	TBARS	Arab	CSS	2.85	1.62	1.34	0.71	7
Ben Mansour 2008 [12]	Tunisia	40	NA	NA	NA	NA	50	NA	NA	CA	African	CC	0.50	0.61	0.12	0.07	6
Tewthanom 2008 [27]	Thailand	20	37.7 ± 13.2	3.4 ± 1.8	NA	4.5 ± 3.3	20	44.9 ± 15.6	NA	MR	Asian	CS	0.65	0.08	0.74	0.10	7
Tewthanom 2008 [27]	Thailand	15	36.3 ± 11.5	13.3 ± 1.3	NA	5.73 ± 6.3	20	44.9 ± 15.6	NA	MR	Asian	CS	0.61	0.06	0.74	0.10	7
Tewthanom 2008 [27]	Thailand	9	39.8 ± 13.6	37.8 ± 8.7	NA	6.6 ± 3.9	20	44.9 ± 15.6	NA	MR	Asian	CS	0.88	0.25	0.74	0.10	7
Turqay 2007 [48]	Turkey	28	35 ± 2.8	NA	NA	NA	11	34 ± 1.7	NA	TBARS	Arab	CSS	1.77	2.17	1.69	0.46	6
Kurien 2003 [49]	USA	35	NA	NA	NA	NA	16	NA	NA	TBARS	Nam	CC	6.97	2.68	4.18	1.21	5
Bae 2002 [50]	Korea	97	34.2 ± 11.3	NA	21.5 ± 3.2	4.5 ± 2.9	97	33.6 ± 10.9	21.6 ± 2.6	FA	Asian	CC	4.14	0.38	3.88	0.28	8
Taysi 2002 [51]	Turkey	24	NA	40.63 ± 8.69	NA	NA	20	NA	NA	TBARS	Arab	CC	9.23	2.61	4.61	0.89	7
Grune 1997 [52]	Germany	11	NA	15.1 ± 5.1	NA	NA	11	13.9 ± 3.7	NA	TBARS	Germans	CS	15.10	5.10	1.53	0.08	7
Grune 1997 [52]	Germany	10	NA	1.8 ± 0.7	NA		11	13.9 ± 3.7	NA	TBARS	Germans	CS	1.80	0.70	1.53	0.08	7
Michel 1997 [53]	Germany	22	15 ± 3	15.1 ± 5.1	NA	1.8 ± 0.7	21	13.8 ± 3.9	NA	TBARS	Germans	CC	1.94	0.18	1.58	0.11	7
Turi 1997 [54]	Hungary	7	NA	NA	NA	NA	15	8.5 ± 3.45	NA	NA	Hungarian	CS	0.24	0.04	0.19	0.02	6
Turi 1997 [54]	Hungary	7	NA	NA	NA	NA	14	32.0 ± 7.03	NA	NA	Hungarian	CS	0.24	0.04	0.20	0.02	6
(b)																	
First author, year	Country	SLE					Control			Assay method, sample	Ethnicity	Study type	ApoB level (SLE)		ApoB level (control)		NOS
		*n*	Age (mean ± sd, years)	SLEDAI	BMI	Duration (mean ± sd, years)	*n*	Age (mean ± sd, years)	BMI				Mean	SD	Mean	SD	

Du 2017 [21]	China	52	31.5 ± 8.86	NA	16 ± 5.31	NA	52	35 ± 7.97	NA	ELISA	Asian	CC	1.08	0.37	0.9	0.15	8
Gaál 2016 [55]	Germany	51	31.82 ± 6.40	24.45 ± 4.13	4 ± 3.05	6.59 ± 5.26	49	31.8 ± 6.81	23.86 ± 4.28	ITA	Germans	CS	0.86	0.26	0.76	0.22	7
Du 2015 [56]	China	16	45 ± 14	NA	6.25 ± 1.98	NA	60	43 ± 8	NA	ELISA	Asian	CS	0.78	0.21	0.59	0.14	7
Du 2015 [56]	China	16	32 ± 15	NA	9.5 ± 3.96	NA	60	43 ± 8	NA	ELISA	Asian	CS	0.79	0.18	0.59	0.14	7
Du 2015 [56]	China	33	36 ± 9	NA	19.5 ± 3.37	NA	60	43 ± 8	NA	ELISA	Asian	CS	1.03	0.31	0.59	0.14	7
Machado 2015 [57]	Brazil	33	15.98 ± 2.0	NA	2.8 ± 3.6	60.25 ± 26.69	33	NA	NA	ELISA	SA	CSS	101.8	33.1	88.3	41	8
Svenungsson 2015 [58]	Sweden	434	46.4 ± 17.55	24.2 ± 4.31	NA	11.43 ± 13.46	322	47.4 ± 17.28	24.63 ± 4.17	ELISA	Sweden	CS	0.82	0.2	0.81	0.23	7
Yuan 2015 [22]	China	46	43.35 ± 9.50	NA	17.75 ± 4.52	NA	40	42.05 ± 9.97	NA	ELISA	Asian	CS	1.1	0.15	0.93	0.13	8
Lilleby 2007 [59]	Norway	57	26.2 ± 9.9	23.8 ± 6	5.5 ± 3.92	10.4 ± 8.2	57	26.3 ± 9.6	24.1 ± 4.7	NA	Caucasian	CS	0.7	0.2	0.7	0.3	8
Sari 2002 [60]	Turkey	24	31.4 ± 9.7	NA	43 ± 7	NA	26	29.7 ± 11.3	NA	NM	Arab	CC	110	22	94	19	7
Formiga 2001 [61]	Spain	53	34.5 ± 7.6	24.1 ± 2.9	4 ± 2	10.5 ± 5.5	45	38.9 ± 2.2	25.9 ± 4.4	RMD	Caucasian	CC	101	30	72	22	7
Ettinger 1988 [62]	USA	28	36 ± 8	25.5 ± 6.3	NA	NA	15	34 ± 9	24.3 ± 4.4	RMD	American	CS	1.16	0.35	0.76	0.22	8
Ettinger 1988 [62]	USA	10	41 ± 12	26 ± 6.6	NA	NA	15	34 ± 9	24.3 ± 4.4	RMD	American	CS	0.82	0.13	0.76	0.22	8

(c)																	
First author, year	Country	SLE					Control			Assay method, sample	Ethnicity	Study type	OxLDL level (SLE)		OxLDL level (control)		NOS
		*n*	Age (mean ± sd, years)	SLEDAI	BMI	Duration (mean ± sd, years)	*n*	Age (mean ± sd, years)	BMI				Mean	SD	Mean	SD	

Ahmad 2014 [63]	Egypt	60	30.8 ± 7.1	26.1 ± 3.2	NA	NA	40	33.4 ± 9.2	25.2 ± 2.8	ELISA	African	CS	1.38	0.79	1.02	0.52	7
Tso 2008 [64]	Taiwan	87	38 ± 2	23 ± 1	4	11	32	40 ± 1	22 ± 1	ELISA	Asian	CC	18.2	2.03	9.62	1.46	7
Huang 2007 [23]	Taiwan	27	NA	NA	NA	NA	32	40 ± 7	22 ± 2	ELISA	Asian	CC	22.6	3.2	9.6	1.5	7
Huang 2007 [23]	Taiwan	31	NA	NA	NA	NA	32	40 ± 7	22 ± 2	ELISA	Asian	CC	25.1	4.7	9.6	1.5	7
McMahon 2006 [65]	USA	154	40.2 ± 13.6	NA	4.5 ± 5.3	10.1 ± 8.7	72	39.9 ± 16.3	NA	ELISA	Mixed	CC	6713.7	3674.5	5356.2	2610	7
Kim 2004 [24]	Korea	72	37.5 ± 10.5	NA	NA	3.4 ± 2.3	39	31.3 ± 6.6	NA	ELISA	Asian	CS	54.3	21.6	40.1	14.2	7
Soep 2004 [25]	USA	33	16.1 ± 4	24.7 ± 7	8 ± 8	3.1 ± 3	30	16.5 ± 4	23.6 ± 5	CA	Mixed	CS	29.7	25.59	19.4	25.59	7

SLE, systemic lupus erythematosus; NA, not available; BMI, body mass index; SLEDAI, systemic lupus erythematosus disease activity index; ELISA, enzyme-linked immunosorbent assay; CC, case-control; CA, colorimetric assay; TBARS, thiobarbituric acid reactive species; MR, microplate reader; FA, fluorometric assay; SMLPO, spectrophotometric method of lipid peroxidase LPO-586; HPLC, high-performance liquid chromatography; NOS, Newcastle–Ottawa Scale; CSS, cross-sectional studies; CS, cohort studies; MDA, malondialdehyde; Nam, North American; SA, South American; ITA, immunoturbidimetric assay; NM, nephelometric method; RMD, radial immunodiffusion; ApoB, apolipoprotein B; OxLDL, oxidized low-density lipoprotein.

**Table 2:** Heterogeneity and publication bias tests.

SLE vs control	df	*P*	SMD (95% CI)	Heterogeneity test		Egger's test		Begg's test	
				*I* ^2^%	*P*	*t*	*P*	Z	*P*
MDA levels	27	<0.001^*∗*^	1.292 (0.780, 1.805)	95.5	<0.001^*∗*^	0.65	0.52	0.97	0.33
ApoB levels	12	<0.001^*∗*^	0.808 (0.451, 1.165)	89.5	<0.001^*∗*^	3.77	≤0.01^*∗*^	1.28	0.20
OxLDL levels	6	<0.001^*∗*^	2.266 (1.088, 3.444)	91.3	<0.001^*∗*^	4.41	0.01^*∗*^	1.95	0.07

^*∗*^Statistical significance (*P* < 0.05). df, degree of freedom; CI, confidence interval.

**Table 3:** Subgroup analysis of MDA, ApoB, and OxLDL levels in SLE.

Oxidants	Subgroups	*N*	SMD (95% CI)	Z	*P*	Heterogeneity test		
						*Q*	*I* ^2^%	*P*
MDA	*Ethnicity*							
	Asian	6	0.317 (−0.575, 1.208)	0.70	0.486	68.27	92.7	<0.001^*∗*^
	Arab	7	2.489 (1.234, 3.743)	3.89	<0.001^*∗*^	269.16	97.8	<0.001^*∗*^
	African	7	0.572 (−0.692, 1.837)	0.89	0.375	188.96	96.8	<0.001^*∗*^
	E/A	8	1.415 (0.785, 2.045)	4.40	<0.001^*∗*^	35.53	80.3	<0.001^*∗*^
	Combined	28	1.292 (0.780, 1.805)	4.94	<0.001^*∗*^	605.64	95.5	<0.001^*∗*^
	*MT*							
	TBARS	13	1.470 (0.470, 2.469)	2.88	0.004^*∗*^	283.23	95.8	<0.001^*∗*^
	ELISA	2	2.355 (1.555, 3.155)	5.77	<0.001^*∗*^	3.83	73.9	0.050^*∗*^
	HPLC	4	1.656 (0.263, 3.049)	2.33	0.020^*∗*^	170.81	98.2	<0.001^*∗*^
	MR	3	−0.559 (−1.898,0.779)	0.82	0.413	19.35	89.7	<0.001^*∗*^
	Others	3	0.717 (0.399, 1.034)	4.42	<0.001^*∗*^	3.37	40.7	0.185
	Combined	26	1.276 (0.722, 1.830)	4.52	<0.001^*∗*^	603.66	96.0	<0.001^*∗*^
	*Age*							
	<36	12	1.304 (0.300, 2.308)	2.54	0.011^*∗*^	272.88	90.0	<0.001^*∗*^
	≥36	12	1.185 (0.353, 2.018)	2.79	0.005^*∗*^	317.90	96.5	<0.001^*∗*^
	Combined	24	1.276 (0.665, 1.886)	4.10	<0.001^*∗*^	591.94	96.1	<0.001^*∗*^
	*SLEDAI*							
	<8	3	0.166 (−1.102, 1.433)	0.26	0.798	16.97	88.2	<0.001^*∗*^
	≥8	15	1.000 (0.222, 1.778)	2.52	0.012^*∗*^	312.20	95.5	<0.001^*∗*^
	Combined	18	0.866 (0.169, 1.563)	2.43	0.015^*∗*^	351.86	95.2	<0.001^*∗*^
	*Duration*							
	<10	11	0.869 (0.255, 1.482)	2.77	0.006^*∗*^	125.16	92.0	<0.001^*∗*^
	≥10	3	2.458 (1.011, 3.905)	3.33	0.001^*∗*^	24.37	91.8	<0.001^*∗*^
	Combined	14	1.202 (0.583, 1.822)	3.80	<0.001^*∗*^	197.16	93.4	<0.001^*∗*^
	*BMI*							
	<23	1	0.779 (0.487, 1.071)	5.23	<0.001^*∗*^	0.00	NA	NA
	≥23	5	1.630 (0.777, 2.482)	3.75	<0.001^*∗*^	39.31	89.8	<0.001^*∗*^
	Combined	6	1.477 (0.749, 2.205)	3.98	<0.001^*∗*^	57.63	91.3	<0.001^*∗*^

OxLDL	*Ethnicity*							
	Asian	4	3.748 (1.225, 6.271)	2.91	0.004^*∗*^	146.31	97.9	<0.001^*∗*^
	African	1	0.518 (0.111, 0.924)	2.5	0.013^*∗*^	0.00	NA	NA
	E/A	2	0.402 (0.157, 0.648)	3.21	0.001^*∗*^	0.00	0.0	1.000
	Combined	7	2.266 (1.088, 3.444)	3.77	<0.001^*∗*^	237.54	97.5	<0.001^*∗*^
	*MT*							
	ELISA	6	2.597 (1.210, 3.984)	3.67	<0.001^*∗*^	231.21	97.8	<0.001^*∗*^
	CA	1	0.403 (−0.097, 0.902)	1.58	0.114	0.00	NA	NA
	Combined	7	2.266 (1.088, 3.444)	3.77	<0.001^*∗*^	237.54	97.5	<0.001^*∗*^
	*Age*							
	<36	3	0.572 (0.324, 0.820)	4.52	<0.001^*∗*^	1.14	0.0	0.567
	≥36	4	3.667 (0.815, 6.518)	2.52	0.012^*∗*^	213.34	98.6	<0.001^*∗*^
	Combined	7	2.266 (1.088, 3.444)	3.77	<0.001^*∗*^	237.54	97.5	<0.001^*∗*^
	*SLEDAI*							
	<8	2	2.451 (−1.590, 6.492)	1.19	0.235	112.59	99.1	<0.001^*∗*^
	≥8	1	0.403 (−0.097, 0.902)	1.58	0.114	0.00	NA	NA
	Combined	3	1.753 (−0.329, 3.836)	1.65	0.099	116.46	98.3	<0.001^*∗*^
	*Duration*							
	<10	2	0.603 (0.285, 0.921)	3.72	<0.001^*∗*^	1.03	2.7	0.311
	≥10	2	2.451 (−1.590, 6.492)	1.19	0.235	112.59	99.1	<0.001^*∗*^
	Combined	4	1.482 (0.128, 2.837)	2.15	0.032^*∗*^	116.68	97.4	<0.001^*∗*^
	*BMI*							
	<23	3	4.681 (4.178, 5.184)	18.3	<0.001^*∗*^	1.79	0.0	0.408
	≥23	2	0.472 (0.157, 0.787)	2.93	0.003^*∗*^	0.12	0.0	0.726
	Combined	5	3.018 (1.016, 5.020)	2.95	0.003^*∗*^	195.17	98.0	<0.001^*∗*^

ApoB	*Ethnicity*							
	Asian	5	1.273 (0.795, 1.751)	5.22	<0.001^*∗*^	18.16	78.0	0.001^*∗*^
	Arab	1	0.781 (0.204, 1.357)	2.66	0.008^*∗*^	0.00	NA	NA
	E/A	7	0.461 (0.109, 0.813)	2.57	0.010^*∗*^	33.38	82.0	<0.001^*∗*^
	Combined	13	0.808 (0.451, 1.165)	4.44	<0.001^*∗*^	114.13	89.5	<0.001^*∗*^
	*MT*							
	ELISA	7	0.958 (0.375, 1.541)	3.22	0.001^*∗*^	91.80	93.5	<0.001^*∗*^
	RMD	3	0.961 (0.481, 1.440)	3.93	<0.001^*∗*^	1.05	44.3	0.166
	Others	2	0.535 (0.198, 0.873)	3.11	0.002^*∗*^	3.59	5.0	0.305
	Combined	12	0.882 (0.496, 1.268)	4.48	<0.001^*∗*^	108.38	89.9	<0.001^*∗*^
	*Age*							
	<36	6	0.521 (0.207, 0.835)	3.25	0.001^*∗*^	13.73	63.6	0.017^*∗*^
	≥36	7	1.034 (0.380, 1.688)	3.10	0.002^*∗*^	100.34	94.0	<0.001^*∗*^
	Combined	13	0.808 (0.451, 1.165)	4.44	<0.001^*∗*^	114.13	89.5	<0.001^*∗*^
	*SLEDAI*							
	<8	5	0.594 (0.145, 1.043)	2.59	0.010^*∗*^	20.61	80.6	<0.001^*∗*^
	≥8	5	1.193 (0.694, 1.691)	4.69	<0.001^*∗*^	19.86	79.9	0.001^*∗*^
	Combined	10	0.891 (0.518, 1.264)	4.68	<0.001^*∗*^	57.51	84.3	<0.001^*∗*^
	*Duration*							
	<10	1	0.415 (0.018, 0.811)	2.05	0.040^*∗*^	0.00	NA	NA
	≥10	4	0.352 (−0.098, 0.801)	1.53	0.125	21.99	86.4	<0.001^*∗*^
	Combined	5	0.359 (−0.005, 0.723)	1.94	0.053	23.55	83.0	<0.001^*∗*^
	*BMI*							
	≥23	5	0.483 (0.077, 0.890)	2.33	0.020^*∗*^	32.97	84.8	<0.001^*∗*^
	Combined	5	0.483 (0.077, 0.890)	2.33	0.020^*∗*^	32.97	84.8	<0.001^*∗*^

^*∗*^Statistical significance (*P* < 0.05). SLE, systemic lupus erythematosus; SLEDAI, systemic lupus erythematosus disease activity index; BMI, body mass index; CA, colorimetric assay; TBARS, thiobarbituric acid reactive species; MR, microplate reader; FA, fluorometric assay; SMLPO, spectrophotometric method of lipid peroxidase LPO-586; HPLC, high-performance liquid chromatography; MDA, malondialdehyde; MT, measurement type; CA, chemiluminescent assay; OxLDL, oxidized low-density lipoprotein; ITA, immunoturbidimetric assay; NM, nephelometric method; RMD, radial immunodiffusion; ApoB, apolipoprotein B; E/A, European/American.

**Table 4:** Results of meta-regression analysis.

Oxidants	Heterogeneity factors	Coefficients	SE	*t*	*P*	95% CI	
						LCI	UCI
MDA	Publication year	−0.052	0.105	−0.49	0.627	−0.267	0.164
	NOS score	0.038	0.857	0.04	0.965	−1.723	1.799
	Sample size	0.005	0.012	0.41	0.686	−0.020	0.030
	Study type	−0.162	1.143	−0.14	0.888	−2.512	2.188
	Country	−0.011	0.464	−0.02	0.981	−0.965	0.943
	Ethnicity	−0.470	0.624	−0.75	0.457	−1.752	0.811
	Measurement type	−0.350	0.650	−0.54	0.596	−1.693	0.994

ApoB	Publication year	0.001	0.018	0.08	0.941	−0.039	0.042
	NOS score	−0.331	0.344	−0.96	0.357	−1.087	0.426
	Sample size	−0.001	0.001	−1.54	0.152	−0.003	0.001
	Study type	−0.144	0.326	−0.44	0.667	−0.860	0.573
	Country	−0.246	0.174	−1.41	0.186	−0.630	0.138
	Ethnicity	−0.510	0.253	−2.02	0.069	−1.066	0.047
	Measurement type	−0.150	0.228	−0.66	0.524	−0.652	0.357

OxLDL	Publication year	0.029	0.301	0.10	0.927	−0.745	0.803
	Sample size	−0.017	0.016	−1.06	0.336	−0.057	0.024
	Study type	−3.076	1.331	−2.31	0.069	−6.497	0.346
	Country	−0.855	0.582	−1.47	0.202	−2.352	0.642
	Ethnicity	−1.238	0.730	−1.70	0.151	−3.112	0.639
	Measurement type	−1.113	1.257	−0.89	0.416	−4.344	2.117

MDA, malondialdehyde; ApoB, apolipoprotein B; OxLDL, oxidized low-density lipoprotein; NOS, Newcastle–Ottawa Scale; CI, confidence interval; LCI, lower confidence interval; UCI, upper confidence interval; SE, standard error.

## Data Availability

The data used to support the findings of this study are available from the corresponding author upon request.
